# Inflammatory indices AISI and SIRI in atherosclerosis risk stratification: validation across community and intensive care populations

**DOI:** 10.1080/07853890.2025.2530792

**Published:** 2025-07-13

**Authors:** Jing Tang, Ting-xin Li, Ling Deng, Xin-cheng Huang, Pei-yuan He, Han Zhao

**Affiliations:** a Department of Health Management Center & Institute of Health Management, Sichuan Provincial People’s Hospital, School of Medicine, University of Electronic Science and Technology of China, Chengdu, China; b Department of Cardiology and Angiology, The Fourth People’s Hospital of Chengdu, Chengdu, China

**Keywords:** Aggregate Index of Systemic Inflammation, Systemic Inflammation Response Index, atherosclerosis, predictive models, restricted cubic spline

## Abstract

**Background:**

Systemic inflammation plays a key role in atherosclerosis development. This study evaluated the predictive performance of two composite inflammatory indices—the Aggregate Index of Systemic Inflammation (AISI) and the Systemic Inflammation Response Index (SIRI)—across community and intensive care populations. The Systemic Immune-Inflammation Index (SII) was also assessed in univariate analysis.

**Methods:**

Data were obtained from a health examination cohort (*n* = 23,516) diagnosed with carotid atherosclerosis *via* ultrasound, and the MIMIC-IV database (*n* = 15,000) classified using ICD codes. Individuals were included based on complete demographic, laboratory, and diagnostic data, with strict exclusion of those with recent acute events or missing values. Logistic regression models were constructed and evaluated with smoothed ROC curves. Non-linear associations were examined using restricted cubic splines (RCS), and subgroup analyses were performed by sex, metabolic status, and ethnicity.

**Results:**

SIRI was consistently associated with atherosclerosis in both cohorts and showed stronger predictive power in men and individuals with metabolic syndrome (*p* < 0.01). AISI showed opposite trends across populations. SII did not show significant associations in univariate analysis and was not included in further modeling. Model performance improved with additional covariates (AUC increased from 0.56 to 0.79). RCS revealed non-linear relationships for both indices. Subgroup effects of SIRI were more prominent in the health examination cohort, while predictive power remained significant across critically ill patients regardless of gender or ethnicity.

**Conclusion:**

SIRI is a robust and consistent predictor of atherosclerosis risk in diverse populations, supporting its utility in routine health assessments and personalized screening. In contrast, AISI’s predictive value is population-dependent. These results support the use of SIRI in personalized screening strategies and suggest its potential utility in routine health assessments.

## Introduction

Atherosclerosis is a chronic inflammatory disease that significantly contributes to cardiovascular morbidity and mortality worldwide [1,2]. Increasing evidence has confirmed that systemic inflammation plays a central role in its initiation and progression by promoting endothelial dysfunction, lipid accumulation, and plaque instability [3–5]. Endothelial dysfunction, characterized by impaired nitric oxide production, increased oxidative stress, and a pro-thrombotic state, not only precedes immune dysregulation but also drives vascular inflammation and plaque progression [6,7]. Inflammatory cells—including neutrophils, monocytes, and lymphocytes—mediate vascular injury and immune balance through cytokine release and oxidative stress, and their imbalance sustains chronic low-grade inflammation leading to atherogenesis [8]. Composite inflammatory markers derived from peripheral blood—such as the Aggregate Index of Systemic Inflammation (AISI) and the Systemic Inflammation Response Index (SIRI)—integrate neutrophil, monocyte, lymphocyte, and platelet counts to reflect this systemic immune activation [9–12]. Compared to traditional indices like the neutrophil-to-lymphocyte ratio (NLR) and the systemic immune-inflammation index (SII), AISI and SIRI may provide broader insights into the inflammatory burden and immune dysregulation associated with atherosclerosis [13] Other composite scores, including the Naples prognostic score, have demonstrated prognostic value in cardiovascular and thromboembolic diseases [14,15], further supporting the role of systemic inflammatory markers in risk stratification.

Although previous studies have reported associations between AISI, SIRI, and cardiovascular outcomes, their predictive value for atherosclerosis across different clinical settings remains underexplored. This study aimed to evaluate and validate the performance of AISI and SIRI in predicting atherosclerosis using a community-based health examination cohort and an intensive care population from the MIMIC-IV database. The findings may support the practical use of these indices in both routine screenings and high-risk patient monitoring.

## Materials and methods

### Ethics statement

This retrospective study adhered to international ethical guidelines and the Declaration of Helsinki [16]. Written informed consent was obtained at the time of health examination, explicitly authorizing data used for research. All data were anonymized. Ethical approval was granted by the Ethics Committee of Sichuan Provincial People’s Hospital (Approval No. 2024-364). Access to MIMIC-IV was obtained by Peiyuan He (ID: 13,494,570) following the completion of CITI training.

### Study population

**Figure 1.**
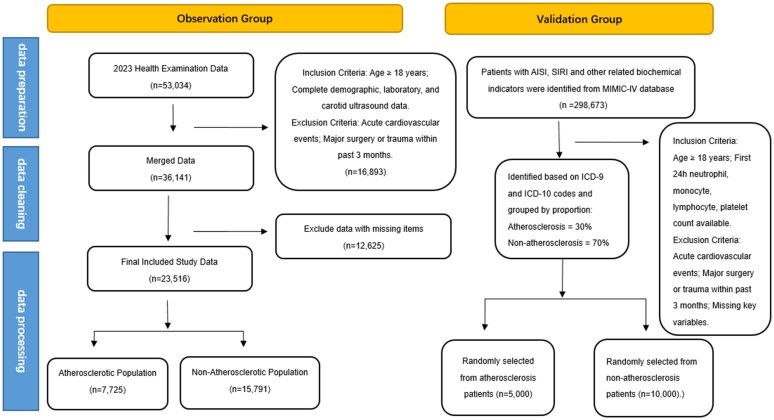
Flowchart of participant selection from the Health Examination Center and MIMIC-IV database. The observation cohort included adults (≥18 years) undergoing carotid Doppler ultrasound in 2023 with complete demographic, laboratory, and ultrasound data. Atherosclerosis was defined as intima-media thickness ≥1.0 mm, plaques, or stenosis. The validation cohort was extracted from the MIMIC-IV database using ICD-9/10 codes. Patients with complete inflammatory cell counts (within 24 h of ICU admission) were included. A total of 5,000 atherosclerosis and 10,000 non-atherosclerosis cases were randomly selected.

The study involved individuals who underwent cervical vascular color Doppler ultrasound at the Health Management Center of Sichuan Provincial People’s Hospital in 2023. Inclusion criteria were: (1) adults aged 18 and older; (2) complete basic demographic information, laboratory tests, and cervical vascular ultrasound results. Exclusion criteria included individuals with acute cardiovascular events, severe trauma, or major surgeries in the past three months, as well as those with missing data. A total of 23,516 subjects were included, comprising 7,725 patients with atherosclerosis and 15,791 without. Data from 298,673 patients were screened from the MIMIC-IV database, categorized into atherosclerosis (30%) and non-atherosclerosis groups (70%) based on ICD-9 and ICD-10 codes. A random sample of 5,000 atherosclerosis patients and 10,000 non-atherosclerosis patients was selected. We included adult patients with available neutrophil, monocyte, lymphocyte, and platelet counts within the first 24 h of ICU admission. Exclusion criteria matched the health examination cohort. The data screening process is shown in Figure 1.

### Data collection

Trained healthcare personnel were responsible for collecting the demographic and health information of the study subjects. Height, weight, and body mass index (BMI) were measured using the HNH-318 weighing scale. Waist circumference (WC) was measured with a tape measure. Blood pressure (systolic blood pressure [SBP] and diastolic blood pressure [DBP]) was measured using an Omron HPB-9020 blood pressure monitor, with the average of two measurements recorded. After fasting for 8 h, venous blood samples were drawn from all study subjects in the morning. Biochemical indicators were uniformly tested at the Sichuan Provincial People’s Hospital Laboratory, including red blood cell count (RBC), hemoglobin (Hb), glycated hemoglobin (HbA1c), glucose (Glu), triglycerides (TG), total cholesterol (TC), low-density lipoprotein cholesterol (LDL-C), high-density lipoprotein cholesterol (HDL-C), cystatin C (Cys C), estimated glomerular filtration rate (eGFR), creatinine (Scr), blood urea nitrogen (BUN), calcium (Ca), uric acid (UA), gamma-glutamyl transferase (GGT), alanine aminotransferase (ALT), aspartate aminotransferase (AST), and counts of neutrophils (NEU), monocytes (MON), lymphocytes (LYM), platelets (PLT), and uric acid (UA).

### Primary outcome and clinical definition

The diagnostic criteria for atherosclerosis differed between the two datasets. In the health examination center, atherosclerosis was diagnosed using color Doppler ultrasound imaging of the carotid and subclavian arteries, conducted with the Philips EPIQ CVx and EPIQ 5 C ultrasound systems. Atherosclerosis was defined as intima-media thickness (IMT) ≥ 1.0 mm or the presence of focal atherosclerotic plaques [17]. In contrast, in the MIMIC-IV database, atherosclerosis was identified based on International Classification of Diseases (ICD-9 and ICD-10) diagnostic codes recorded in electronic medical records.

Definition of Inflammatory Indices and Metabolic Syndrome Criteria:

1. Aggregate Index of Systemic Inflammation (AISI)

AISI was calculated using the formula:

AISI=(Neutrophil count×Monocyte count×Platelet count)/Lymphocyte count.

This formula integrates three key pro-inflammatory cell types (neutrophils, monocytes, and platelets) relative to lymphocytes.

Systemic Inflammatory Response Index (SIRI)

SIRI was calculated using the formula:

SIRI=(Neutrophil count×Monocyte count)/Lymphocyte count.

Both indices were analyzed as exposure variables in the predictive models.

Systemic immune-inflammation Index (SII)

SII was calculated using the formula:

SII=(Neutrophil count × Platelet count)/Lymphocyte count.

Naples score was not calculated due to dataset limitations and is discussed only for background comparison.

Additionally, the criteria for diagnosing metabolic syndrome (MetS) were based on the International Diabetes Federation (IDF) standards established in 2005 [18] Central (abdominal) obesity was a required condition, defined for the Asian population as a waist circumference of ≥90 cm in men and ≥80 cm in women. In addition, at least two of the following criteria had to be met: (1) triglycerides (TG) ≥ 1.7 mmol/L or ongoing treatment for elevated TG; (2) HDL-C < 1.03 mmol/L in men or <1.29 mmol/L in women, or specific treatment; (3) blood pressure ≥130/85 mmHg or use of antihypertensive medication; (4) fasting plasma glucose ≥5.6 mmol/L or a diagnosis of diabetes.

### Statistical analysis

In this study, baseline characteristics were first analyzed using SPSS version 25.0. For continuous variables, the Mann-Whitney U test was employed to assess differences between the two groups, with results presented as median and interquartile range [*M(P_25_−P_75_*)]. Categorical variables were analyzed using the chi-square test (*χ*^2^ test) and described in terms of frequency and percentage [*n* (%)]. Subsequently, univariate logistic regression analysis was conducted using R version 4.0.5 to evaluate the impact of each independent variable on atherosclerosis. To identify the optimal model fitting approach, stepwise regression (backward method) was utilized to eliminate insignificant variables, ultimately constructing a multivariate regression model. Model interpretation was assessed through smoothed receiver operating characteristic (sROC) curve analysis, with the area under the curve (AUC) reported to quantify the model’s discriminatory ability. Additionally, restricted cubic spline (RCS) analysis was performed to explore potential nonlinear relationships between independent variables and atherosclerosis. Finally, subgroup interaction effects were analyzed to clarify the differential impact of variables across different subgroups. Throughout the model construction and analysis process, external validation was conducted to ensure the robustness and generalizability of the results.

## Results

### Baseline characteristics based on Health Examination Center data

The AISI was significantly lower in the atherosclerosis group [196.43 (163.04–233.31)] compared to the non-atherosclerosis group [208.70 (174.10–246.10), *p* < 0.01], while the SIRI was higher [0.71 (0.50–1.03) *vs.* 0.63 (0.45–0.90), *p* < 0.01]. Conversely, the SII was slightly lower in the atherosclerosis group [366.60 (270.06–500.55)] than in the non-atherosclerosis group [370.36 (275.45–502.07), *p* < 0.01], showing a trend consistent with AISI but opposite to SIRI. Other variables, including age, sex distribution, and metabolic syndrome, also differed significantly between groups (Table 1).

**Table 1.** Baseline characteristics based on Health Examination Center data.

Variable	Over all (*n* = 23,516)	Atherosclerosis (*n* = 7725)	No atherosclerosis (*n* = 15,791)	*Z*/*X*^2^ value	*p*-Value
Age	52.00 (42.00–60.00)	59.00 (52.00–68.00)	48.00 (39.00–55.00)	67.43	<0.01
Weight (kg)	66.00 (57.00–74.00)	66.00 (58.00–74.00)	65.40 (57.00–74.00)	4.14	<0.01
Gender					
Male^a^	14,888 (63.31)	5,406 (69.98)	9,482 (60.05)	220.37	<0.01
Female^a^	8,628 (36.69)	2,319 (30.02)	6,309 (39.95)		
Biochemical indicators					
AISI	204.31 (170.39–242.39)	196.43 (163.04–233.31)	208.70 (174.10–246.10)	15.62	<0.01
SIRI	0.66 (0.46–0.94)	0.71 (0.50–1.03)	0.63 (0.45–0.90)	15.53	<0.01
SII	370.36 (275.45–502.07)	366.60 (270.06–500.55)	371.73 (277.58–502.45)	6.97	<0.01
SBP (mmHg)	121.00 (110.00–134.00)	128.00 (116.00–140.50)	119.00 (108.00–130.00)	35.69	<0.01
DBP (mmHg)	74.00 (67.00–83.00)	76.00 (68.00–84.00)	73.00 (66.00–82.00)	15.53	<0.01
WC (cm)	83.00 (76.00–90.00)	86.00 (79.00–92.00)	82.00 (74.00–89.00)	26.25	<0.01
BMI (kg/m²)	24.24 (22.15–26.37)	24.61 (22.66–26.64)	24.02 (21.94–26.22)	12.71	<0.01
RBC (10^12/L)	4.80 (4.45–5.14)	4.76 (4.44–5.08)	4.81 (4.46–5.16)	7.31	<0.01
Hb (g/dL)	147.00 (135.00–158.00)	147.00 (136.00–157.00)	147.00 (135.00–158.00)	0.14	0.89
HbA1c (%)	5.60 (5.34–5.90)	5.79 (5.49–6.15)	5.54 (5.30–5.81)	38.15	<0.01
GLU (mmol/L)	4.87 (4.52–5.36)	5.06 (4.66–5.78)	4.79 (4.46–5.21)	31.22	<0.01
TG (mmol/L)	1.39 (0.98–2.04)	1.46 (1.06–2.10)	1.35 (0.94–2.02)	10.40	<0.01
TC (mmol/L)	4.82 (4.22–5.45)	4.91 (4.24–5.58)	4.78 (4.22–5.39)	7.33	<0.01
LDL-C (mmol/L)	2.91 (2.37–3.44)	2.98 (2.36–3.55)	2.88 (2.38–3.39)	6.68	<0.01
HDL-C (mmol/L)	1.28 (1.08–1.55)	1.26 (1.06–1.51)	1.30 (1.09–1.57)	7.93	<0.01
CysC (mg/L)	0.88 (0.80–0.98)	0.94 (0.85–1.05)	0.86 (0.78–0.95)	41.69	<0.01
eGFR (mL/min/1.73 m²)	91.20 (80.50–101.20)	86.10 (74.9–95.70)	93.90 (83.30–103.60)	38.76	<0.01
Cr (μmol/L)	78.90 (67.00–89.80)	80.40 (68.9–91.00)	78.00 (65.90–89.10)	10.88	<0.01
BUN (mmol/L)	5.15 (4.38–6.07)	5.39 (4.58–6.37)	5.05 (4.29–5.92)	19.20	<0.01
Ca (mg/dL)	2.36 (2.31–2.42)	2.36 (2.30–2.42)	2.37 (2.31–2.42)	3.25	<0.01
UA (μmol/L)	351.00 (291.00–414.00)	354.00 (297.00–414.00)	350.00 (288.00–414.00)	3.93	<0.01
GGT (U/L)	24.00 (16.00–41.00)	25.00 (17.00–41.00)	23.00 (15.00–40.00)	8.91	<0.01
ALT (U/L)	20.00 (14.00–30.00)	20.00 (15.00–29.00)	20.00 (14.00–30.00)	0.08	0.94
AST (U/L)	20.00 (17.00–25.00)	21.00 (18.00–25.00)	20.00 (17.00–25.00)	7.35	<0.01
Alcohol history					
Non-drinker^a^	14,677 (62.41)	4,832 (62.55)	9,934 (62.91)	36.78	<0.01
Occasional^a^	6,563 (27.91)	2,055 (26.60)	4,508 (28.55)		
Regular^a^	2,187 (9.30)	838 (10.85)	1,394 (8.83)		
Smoking status					
Non-smoker^a^	19,143 (81.40)	5,995 (77.61)	13,148 (83.26)	109.68	<0.01
Smoker^a^	4,373 (18.60)	1,730 (22.39)	2,643 (16.74)		
Metabolic syndrome					
Yes^a^	5,879 (25.00)	2,575 (33.33)	3,304 (20.92)	426.08	<0.01
No^a^	17,637 (75.00)	5,150 (66.67)	12,487 (79.08)		

AISI: Total Systemic Inflammation Index; SIRI: Systemic Inflammatory Response Index; SII: Systemic Immune-Inflammation Index; SBP: systolic blood pressure; DBP: diastolic blood pressure; WC: waist circumference; BMI: body mass index; RBC: red blood cell count; Hb: hemoglobin; HbA1c: glycated hemoglobin; GLU: glucose; TG: triglycerides; TC: total cholesterol; LDL-C: low-density lipoprotein cholesterol; HDL-C: high-density lipoprotein cholesterol; CysC: cystatin C; eGFR: estimated glomerular filtration rate; Cr: creatinine; BUN: blood urea nitrogen; Ca: calcium; UA: uric acid; GGT: gamma-glutamyl transferase; ALT: alanine aminotransferase; AST: aspartate aminotransferase.

Data were presented as *M(P_25_-P_75_)*.

^a^The data outside the brackets are the number of cases, the data inside the brackets are the constituent ratio (%).

### Univariate regression analysis based on Health Examination Center data

Logistic regression revealed that SIRI was positively associated with atherosclerosis (OR = 1.36, *p* < 0.01), while AISI (OR = 1.00, *p* < 0.01) and SII (OR = 1.00, *p* = 0.07) showed limited or inconsistent associations. Additional significant predictors included CysC (OR = 34.41, *p* < 0.01), HbA1c (OR = 1.74, *p* < 0.01), and metabolic syndrome (OR = 1.89, *p* < 0.01), exhibited significant positive correlations. Age (OR = 0.64, *p* < 0.01) and RBC (OR = 0.81, *p* < 0.01) were negatively associated with atherosclerosis risk (Table 2).

**Table 2.** Univariate logistic regression based on Health Examination Center data.

Variable	*OR*	*p*-Value
AISI	1.00 (1.00–1.01)	<0.01
SIRI	1.36 (1.29–1.43)	<0.01
SII	1.00 (1.00–1.00)	0.07
Age	0.64 (0.61–0.68)	<0.01
Gender	1.09 (1.09–1.09)	<0.01
Weight	1.00 (1.00–1.01)	<0.01
SBP	1.03 (1.03–1.03)	<0.01
DBP	1.02 (1.02–1.02)	<0.01
WC	1.04 (1.03–1.04)	<0.01
BMI	1.05 (1.04–1.06)	<0.01
RBC	0.81 (0.77–0.86)	<0.01
Hb	1.00 (1.00–1.01)	0.83
HbA1c	1.74 (1.68–1.81)	<0.01
GLU	1.33 (1.31–1.36)	<0.01
TG	1.03 (1.01–1.04)	<0.01
TC	1.10 (1.07–1.14)	<0.01
LDL-C	1.11 (1.07–1.15)	<0.01
HDL-C	0.74 (0.69–0.80)	<0.01
CysC	34.41 (28.64–41.42)	<0.01
eGFR	0.96 (0.96–0.96)	<0.01
Cr	1.01 (1.01–1.01)	<0.01
BUN	1.23 (1.2–1.25)	<0.01
Ca	0.63 (0.47–0.86)	<0.01
UA	1.00 (1.00–1.01)	<0.01
GGT	1.00 (1.00–1.01)	<0.01
ALT	1.00 (1.00–1.01)	<0.01
AST	1.00 (1.00–1.01)	<0.01
Alcohol history	1.06 (1.02–1.11)	<0.01
Smoking status	1.44 (1.34–1.54)	<0.01
Metabolic syndrome	1.89 (1.78–2.01)	<0.01

AISI: Total Systemic Inflammation Index; SIRI: Systemic Inflammatory Response Index; SII: Systemic Immune-Inflammation Index; SBP: systolic blood pressure; DBP: diastolic blood pressure; WC: waist circumference; BMI: body mass index; RBC: red blood cell count; Hb: hemoglobin; HbA1c: glycated hemoglobin; GLU: glucose; TG: triglycerides; TC: total cholesterol; LDL-C: low-density lipoprotein cholesterol; HDL-C: high-density lipoprotein cholesterol; CysC: cystatin C; eGFR: estimated glomerular filtration rate; Cr: creatinine; BUN: blood urea nitrogen; Ca: calcium; UA: uric acid; GGT: gamma-glutamyl transferase; ALT: alanine aminotransferase; AST: aspartate aminotransferase.

### Stepwise regression based on Health Examination Center data

Backward stepwise regression identified an optimal predictive model incorporating inflammatory indices and covariates including Age, Gender, Weight, DBP, WC, RBC, HbA1c, TG, TC, HDL-C, CysC, eGFR, Scr, BUN, alcohol history, smoking status, and MetS. The final model demonstrated improved fit (AIC reduced from 23,924.00 to 23,909.94), supporting the incremental predictive value of SIRI and AISI when combined with metabolic and clinical variables.

### Multi-factor regression model construction based on two databases

This study, based on the multifactorial regression analysis results from the health examination center and the MIMIC-IV database, revealed differences in the performance of AISI and SIRI.

AISI: In the health checkup data, higher AISI levels were inversely associated with atherosclerosis risk (T4 *vs.* T1: OR = 0.58, *p* < 0.01), while in the MIMIC-IV data, elevated AISI significantly increased risk (T4 *vs.* T1: OR = 3.27 in Model 1; OR = 3.56 in Model 2, both *p* < 0.01).

SIRI: Consistent positive associations were shown across both datasets. In the health examination cohort, the ORs for T2–T4 *vs.* T1 were 1.20, 1.38, and 1.75, respectively (*p* < 0.01). In the MIMIC-IV data, SIRI showed even stronger associations: T4 *vs.* T1 OR = 9.28 in Model 1 and OR = 5.04 in Model 2 (*p* < 0.01) (Table 3).

**Table 3.** Associations of AISI and SIRI with clinical outcomes: multivariate analysis from two independent databases.

Groups	Model	AISI		SIRI	
		Health Exam Center OR (95% *CI*), *p*	MIMIC-IV OR (95% *CI*), *p*	Health Exam Center OR (95% *CI*), *p*	MIMIC-IV OR (95% *CI*), *p*
All	Model 1	1.00 (1.00–1.00), <0.01	1.00 (1.00–1.00), <0.01	1.36 (1.29–1.43), <0.01	1.06 (1.06–1.07), <0.01
	Model 2	1.00 (1.00–1.00), 0.04	1.00 (1.00–1.00), <0.01	1.00 (0.93–1.08), 0.02	1.03 (1.03–1.04), <0.01
	Model 3	1.00 (1.00–1.00), 0.05	–	0.98 (0.90–1.06), 0.61	–
	Model 4	1.00 (1.00–1.00), 0.71	–	0.99 (0.91–1.07), 0.77	–
T1	Reference				
T2	Model 1	0.82 (0.76–0.88), <0.01	1.18 (1.06–1.32), <0.01	1.20 (1.11–1.30), <0.01	3.54 (3.11–4.03), <0.01
	Model 2	1.06 (0.97–1.15), 0.19	1.50 (1.32–1.70), <0.01	1.11 (1.01–1.21), 0.03	2.53 (2.20–2.90), <0.01
	Model 3	1.08 (0.99–1.17), 0.09	–	1.09 (1.00–1.19), 0.06	–
	Model 4	1.05 (0.96–1.14), 0.31	–	1.06 (0.97–1.16), 0.21	–
T3	Model 1	0.68 (0.63–0.73), <0.01	1.87 (1.66–2.11), <0.01	1.38 (1.28–1.50), <0.01	6.97 (6.16–7.91), <0.01
	Model 2	1.05 (0.97–1.15), 0.25	2.35 (2.05–2.69), <0.01	1.13 (1.03–1.24), <0.01	4.07 (3.57–4.66), <0.01
	Model 3	1.07 (0.98–1.16), 0.14	–	1.10 (1.00–1.21), 0.05	–
	Model 4	1.01 (0.92–1.10), 0.86	–	1.05 (0.95–1.16), 0.34	–
T4	Model 1	0.58 (0.54–0.63), <0.01	3.27 (2.75–3.89), <0.01	1.75 (1.62–1.90), <0.01	9.28 (8.16–10.57), <0.01
	Model 2	1.10 (1.01–1.21), 0.03	3.56 (2.93–4.32), <0.01	1.22 (1.11–1.33), <0.01	5.04 (4.40–5.78), <0.01
	Model 3	1.10 (1.00–1.20), 0.04	–	1.16 (1.03–1.32), 0.02	–
	Model 4	1.00 (0.92–1.10), 0.95	–	1.09 (0.96–1.23), 0.20	–

AISI: Aggregate Index of Systemic Inflammation; Physical examination center: ALL (MID + IQR) = 204.31 (170.40–242.38); T1: ≤170.40; T2: >170.40 and ≤204.31; T3: >204.31 and ≤242.38; T4: >242.38. MIMIC-IV database: ALL (MID + IQR) = 326.66 (272.83–402.38); T1: ≤272.83; T2: >272.83 and ≤326.66; T3: >326.66 and ≤402.38; T4: >402.38. SIRI: Systemic Inflammation Response Index; Physical Examination Center: ALL (MID + IQR): 0.66 (0.46–0.94); T1: ≤0.46; T2: >0.46 and ≤0.66; T3: >0.66 and ≤0.94; T4: >0.94; MIMIC-IV Database: ALL (MID + IQR): 2.56 (1.30–5.14); T1: ≤1.30; T2: >1.30 and ≤2.56; T3: >2.56 and ≤5.14; T4: >5.14. Model 1: Inflammatory index. Model 2: Model 1 + age + gender. Model 3: Model 2 + glomerular filtration rate + urea + cystatin C. Model 4: Model 3 + diastolic blood pressure + waist circumference + body weight + red blood cell count + glycated hemoglobin + triglycerides + total cholesterol + high-density lipoprotein + alcohol consumption history + smoking status + metabolic syndrome.

### Interpretability results of sROC model based on two databases

**Figure 2.**
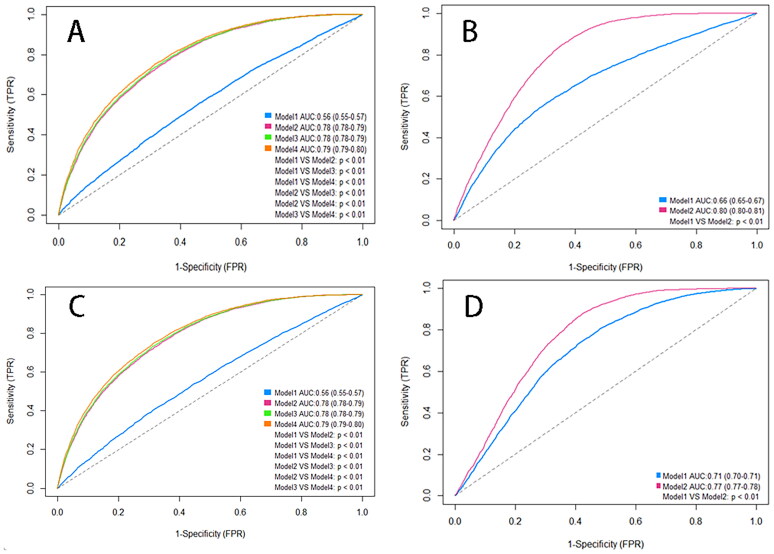
Combined sROC analysis of SIRI and AISI in health examination and MIMIC-IV databases. Panel organization: (A,C) Health Examination Center cohort; (B,D) MIMIC-IV database. Within each panel: upper curve/row: Systemic Inflammation Response Index (SIRI); lower curve/row: Aggregate Index of Systemic Inflammation (AISI). Model specifications: Model 1: Inflammatory Index; Model 2: Model 1 + Age + Gender; Model 3: Model 2 + Glomerular Filtration Rate + Urea + Cystatin C; Model 4: Model 3 + Diastolic Blood Pressure + Waist Circumference + Body Weight + Red Blood Cell Count + Glycated Hemoglobin + Triglycerides + Total Cholesterol + High-Density Lipoprotein + Alcohol Consumption History + Smoking Status + Metabolic Syndrome. *Note:* All models were multivariate logistic regression analyses estimating ORs (95% CIs) for atherosclerosis risk across index quartiles (T1–T4).

In the health examination cohort, AUCs for AISI and SIRI in Model 1 were modest (0.56). Performance improved markedly in Model 2 (AUC = 0.78) and Model 3 (AUC = 0.79), indicating the value of integrating additional predictors. In MIMIC-IV, AUCs for AISI were 0.66 (Model 1) and 0.80 (Model 2); for SIRI, 0.71 (Model 1) and 0.77 (Model 2), all *p* < 0.01, supporting the robustness of multi-variable models (Figure 2).

### Restricted cubic spline analysis based on two databases

The restricted cubic spline plots illustrate the nonlinear associations between AISI and SIRI with the risk of atherosclerosis. Initially, Models 1, 2, and 3 were calculated using data from the health examination center, followed by validation of Models 1 and 2 using the MIMIC-IV database; Model 4 was excluded from analysis due to non-significant results.

Model 1: In the health examination cohort, AISI showed a decreasing trend in odds ratios beyond the inflection point of 204.50. In contrast, in the MIMIC-IV cohort, AISI exhibited an increasing trend after 304.13. SIRI demonstrated a consistently increasing trend in both cohorts, with inflection points at 0.66 (health exam) and 3.05 (MIMIC-IV).

Model 2: AISI showed an increasing trend beyond 204.50 in the health examination cohort, and a more pronounced rise after 304.13 in the MIMIC-IV cohort. SIRI exhibited a decreasing trend in both cohorts, with inflection points at 0.63 (health exam) and 3.05 (MIMIC-IV).

**Figure 3.**
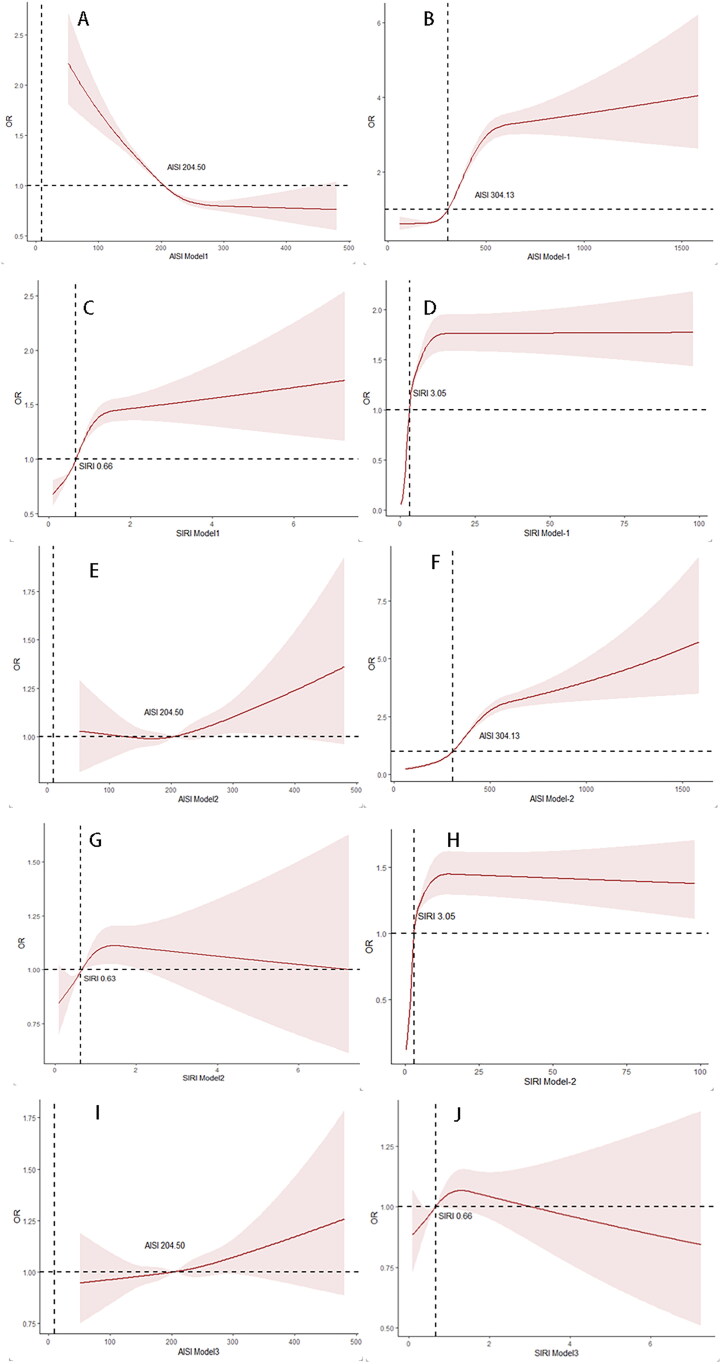
Restricted cubic spline plots for the association of AISI and SIRI with atherosclerosis risk across three multivariate models. Restricted cubic spline (RCS) plots demonstrating the nonlinear association of the Aggregate Index of Systemic Inflammation (AISI) and the Systemic Inflammation Response Index (SIRI) with atherosclerosis risk. Each subplot shows odds ratios (solid lines) and 95% confidence intervals (shaded areas), with the median index value as the reference. The left panels represent data from the Health Examination Center, and the right panels represent data from the MIMIC-IV database. (A–D) (Top row): Model 1—Crude model using inflammatory index only. (E–H) (Middle row): Model 2—Adjusted for age and gender. (I,J) (Bottom row): Model 3—Adjusted for age, gender, glomerular filtration rate, urea, and cystatin C (Health Examination Center only). AISI: Aggregate Index of Systemic Inflammation; SIRI: Systemic Inflammation Response Index; RCS: restricted cubic spline.

Model 3 (health examination cohort only): AISI again showed an increasing trend beyond 204.50, while SIRI demonstrated a more significant decline in risk beyond 0.66 (Figure 3).

### Analysis of subgroup interaction effects based on two databases

Subgroup interaction analysis revealed differences in the predictive effects of AISI and SIRI across various subgroups:

AISI showed no significant interactions with age, sex, smoking/drinking status, or MetS in either cohort.

SIRI showed stronger predictive value in men and individuals with MetS in the health checkup cohort (OR = 1.38, *p* < 0.01). In MIMIC-IV, SIRI remained predictive across different age groups, sexes, and ethnicities, although OR variations were modest (Figure S1).

## Discussion

This study revealed significantly elevated SIRI levels in atherosclerotic patients, with particularly pronounced differences observed in the health examination cohort. In contrast, AISI levels were lower in atherosclerotic patients compared to non-atherosclerotic individuals. This finding contradicts previous studies based on health examination center data [19], indicating a notable difference in systemic inflammation levels and reflecting the complexity of inflammatory mechanisms. The inconsistent performance of AISI may reflect the population-specific variability of its components—particularly platelets and monocytes—and their context-dependent behavior in different clinical settings. Such fluctuations may undermine AISI’s utility as a universal inflammatory marker for atherosclerosis risk stratification.

As an upstream trigger and downstream target of immune dysregulation, endothelial dysfunction represents a central pathophysiological mechanism linking systemic inflammation and atherosclerosis. It can initiate and amplify inflammatory cascades and is influenced by comorbidities, age, and disease severity. Given this, differences in endothelial status across cohorts may partly explain the opposite direction of AISI associations, especially between community and ICU populations. Future studies incorporating endothelial biomarkers may improve the interpretability and predictive performance of AISI and related indices.

Baseline characteristic analysis revealed significant differences in factors, such as age [20], gender [21], and MetS [22,23], which were also mentioned in earlier research, further emphasizing the multifactorial pathological mechanisms underlying atherosclerosis. Interestingly, in the health examination cohort, age showed a negative association with atherosclerosis in univariate analysis. This counterintuitive result may be due to selection bias, as elderly individuals with known or suspected cardiovascular risk are more likely to undergo carotid ultrasound screening during health checkups. Such bias could result in overrepresentation of relatively healthier older adults in the non-atherosclerosis group, thus producing a non-classical statistical trend.

Univariate regression analysis demonstrated a significant association between SIRI and the risk of atherosclerosis, with increased levels markedly elevating the disease risk. This finding is consistent with studies on cardiovascular mortality risk [24] and aligns with research on cardiovascular disease risk in rural populations [25]. In contrast, AISI did not show a significant association. Other factors, such as HbA1c [26], MetS, and CysC [27,28], were also significantly associated with atherosclerosis, reinforcing the critical role of systemic inflammation and metabolic abnormalities in the development of this condition. Although SII was included in baseline and univariate analyses, it was excluded from further modeling due to its limited discriminatory capacity in sROC and RCS assessments.

The optimized model constructed through stepwise regression analysis indicated that both AISI and SIRI demonstrate predictive efficacy for the risk of atherosclerosis. Notably, variables, such as MetS, CysC, and WC significantly enhanced the model’s predictive capability. This finding aligns with previous research suggesting that CysC serves as a potential biochemical marker for the early diagnosis of atherosclerosis [29], as well as studies indicating a correlation between WC in elderly women and young men with carotid artery thickness [30,31].

Multivariable regression analysis revealed discrepancies in the performance of AISI and SIRI between the health examination center data and the MIMIC-IV database. In the health examination center data, higher AISI levels were negatively correlated with atherosclerosis risk, whereas in the MIMIC-IV database, they were associated with an increased risk. This highlights that AISI’s predictive capacity may be modified by differences in baseline inflammatory states and comorbid conditions across populations. Previous studies have indicated a significant association between elevated AISI and cardiovascular mortality risk in patients with acute myocardial infarction [32]. In contrast, SIRI exhibited consistent performance across both databases, demonstrating its substantial stability and sensitivity as a predictor of atherosclerosis risk [33]. Additionally, research has shown that SIRI can serve as an independent predictor of coronary heart disease in individuals with non-alcoholic fatty liver disease [34], suggesting its predictive capacity is relatively stable and less influenced by other factors.

sROC curve analysis indicated that the predictive capacity of AISI or SIRI alone is limited, consistent with previous studies reporting an AUC of 0.644 for SIRI [35]. However, as additional variables were incorporated into the model, the predictive performance improved significantly, with the final model achieving an AUC of 0.79. This finding suggests that combining AISI and SIRI with other metabolic and clinical indicators enhances predictive accuracy.

Restrictive cubic spline analysis revealed a non-linear relationship between AISI and SIRI and the risk of atherosclerosis. AISI exhibited varying non-linear patterns across different models, indicating that its predictive efficacy is influenced by multiple factors. After controlling for confounding variables, the predictive ability of AISI for atherosclerosis risk became more pronounced. In contrast, the relationship between SIRI and atherosclerosis risk displayed opposing trends across different models, suggesting its complexity in risk assessment. While previous studies did not directly establish a link between atherosclerosis and SIRI [36], other research has indicated a non-linear relationship between elevated SIRI levels and hypertension prevalence, as well as adverse effects of high SIRI on the vulnerability and burden of intracranial plaques [37].

Subgroup analysis indicated that SIRI demonstrated a more pronounced predictive effect in men and patients with metabolic syndrome, particularly in high-risk subgroups, where its predictive value was notably stable. This finding aligns with the conclusions of Yan Chen et al. [38] suggesting that systemic inflammation is more closely related to the pathogenesis of atherosclerosis in these specific populations.

Despite demonstrating the potential of AISI and SIRI in predicting atherosclerosis risk, this study has several limitations. While utilizing two large databases enhances robustness, the diversity of study subjects may limit generalizability, and the cross-sectional design precludes causal inferences—highlighting the need for longitudinal validation. Although sROC curves and stepwise regression improved predictive capability, variable interactions remain underexplored, and further analyses are warranted. Furthermore, the differing definitions of atherosclerosis across the two databases (imaging-based *vs.* ICD coding) may have introduced classification bias, limiting direct comparability and requiring cautious interpretation of inter-cohort findings.

This study highlights SIRI as a robust predictor of atherosclerosis across both general and critically ill populations. Given its biological plausibility, affordability, and consistent performance, we recommend incorporating SIRI into routine screening protocols, especially for high-risk groups, such as men and those with MetS. AISI, though less stable, may still contribute valuable information when used in combination with other indicators.

Future studies should focus on longitudinal and interventional designs to (1) validate causality, (2) explore marker interactions, and (3) optimize multi-marker models for improved clinical stratification. Particular attention should be paid to the role of inflammatory indices in subgroups defined by sex, metabolic status, and age.

## Conclusion

SIRI showed stable and significant predictive value for atherosclerosis across both community and ICU populations, particularly in males and individuals with metabolic syndrome. In contrast, AISI exhibited inconsistent associations, indicating population-dependent variability. Although both indices alone had moderate predictive performance, combining them with metabolic and renal markers substantially improved model accuracy. Nonlinear patterns observed in spline analysis further support the complex role of inflammation in atherosclerosis. Given its consistency and practicality, SIRI is recommended as a routine marker for cardiovascular risk screening in high-risk groups. AISI may provide complementary value when integrated into multi-marker models.

## Supplementary Material

Supplemental Material

## Data Availability

This study analyzed two datasets. One publicly available dataset was obtained from the MIMIC-IV database, which can be accessed at https://mimic.physionet.org/. The other dataset, analyzed from Sichuan Provincial People’s Hospital, is not publicly available but can be obtained from Han Zhao (bryant_neo@163.com) upon reasonable request.
