# Supplementary material for: Inflammatory indices AISI and SIRI in atherosclerosis risk stratification: validation across community and intensive care populations
Source: Ann Med. 2025 Jul 13;57(1):2530792. doi: 10.1080/07853890.2025.2530792 (PMC12258198; doi:10.1080/07853890.2025.2530792)

Supplementary Figure1 Subgroup Interaction Effects of AISI and SIRI in Two Independent Cohorts

Panels A–D show subgroup interaction effects of AISI and SIRI across two populations. The left panels represent results from the Health Examination Center, and the right panels from the MIMIC-IV database. The top panels show interactions based on AISI, and the bottom panels show those based on SIRI. Subgroups analyzed include age, gender, metabolic syndrome, hypertension, diabetes, and dyslipidemia. Interaction terms were tested using multiplicative models. P values for interaction are shown. Abbreviations: AISI, Aggregate Index of Systemic Inflammation; SIRI, Systemic Inflammation Response Index.


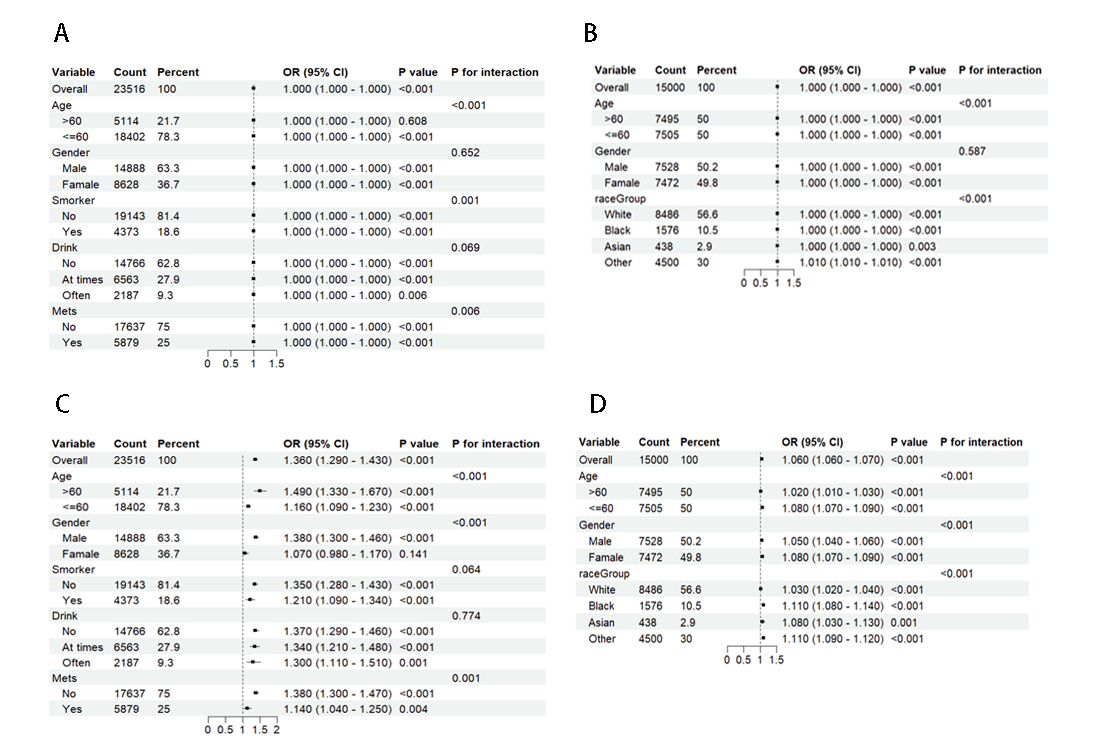

Supplement: Supplemental Material [file IANN_A_2530792_SM0325.docx]
